# Real world duration of curative intent breast, colorectal, non-small cell lung, and prostate cancer treatment

**DOI:** 10.1186/s12885-021-07923-4

**Published:** 2021-03-02

**Authors:** Selina K. Wong, Jeremy Hamm, Aria Shokoohi, Colleen E. McGahan, Cheryl Ho

**Affiliations:** 1 Department of Medical Oncology, BC Cancer, Vancouver, BC Canada; 2 University of British Columbia, Vancouver, BC Canada; 3 Department of Cancer Surveillance and Outcomes, BC Cancer, Vancouver, BC Canada

**Keywords:** Adjuvant, Treatment duration, Medical leave, Breast, Colorectal, Workplace, Financial

## Abstract

**Background:**

Advances in curative treatment for breast, colorectal, NSCLC and prostate cancer have led to improvements in cancer survival. Cancer treatment and recovery time can vary depending on the recommended modalities and intensity of therapy. Our objective was to determine the current real world duration of curative treatments for the four common cancers.

**Methods:**

A retrospective review was completed of patients referred to BC Cancer from 2010 to 2016, ≤ 65 years old, newly diagnosed with stage I-III breast, colorectal, NSCLC or prostate cancer who received curative intent treatment. Information was collected on baseline characteristics, date of diagnosis, surgery, type, duration and intent of both radiotherapy and chemotherapy.

**Results:**

In total, 22,275 patients were included: 55.7% breast, 22.4% colorectal, 9.2% NSCLC, 12.7% prostate cancer. Stage I/II/III at diagnosis: breast 47.2/38.7/14.1%, colorectal 26.5/30.1/43.5%, NSCLC 46.5/18.1/35.4%, prostate 7.7/62.9/29.4%. Patients treated with definitive surgery only: breast 35.9%, colorectal 58%, NSCLC 52.2%, prostate 40.1%. The median duration of multimodality treatment was breast 24.6 weeks, colorectal 26.7 weeks, NSCLC 9.1 weeks, and prostate 6.0 weeks.

**Conclusions:**

Approximately half of patients who undergo curative cancer treatment require definitive radiotherapy or multimodality treatment. The median duration of therapy for the most commonly treated cancers ranged from 6.0–26.7 weeks. Multimodality curative treatment can be prolonged for selected cancers when accounting for the duration of adjuvant chemotherapy and radiotherapy and recovery time between modalities.

## Background

Across the most commonly diagnosed cancers, breast, colorectal, non small cell lung cancer (NSCLC), and prostate, advances in multimodality treatments have led to improvements in cancer survival. Breast cancer has benefited from the judicious use of chemotherapy, radiotherapy and hormone treatment in a variety of sequences to improve outcomes [1, 2]. Similarly, the use of adjuvant 5 fluorouracil and oxaliplatin-based regimens has improved cure rates with colorectal cancer [3, 4]. For non-small cell lung cancer (NSCLC), adjuvant chemotherapy is the current standard of care for resected early stage disease as is consolidation durvalumab after chemoradiotherapy for stage III disease [5, 6]. Prostate cancer guidelines summarize who should be considered for adjuvant or definitive radiotherapy [7].

Guidelines and protocols can provide estimates of therapy duration for chemotherapy and radiotherapy however, they do not provide insight into the recovery time needed between treatments. Understanding the full duration of therapy is important for patients to arrange their support systems and to notify their employers. Cancer treatments may require reduction in employment commitments and/or workplace leaves. Various countries have different government funded supports for sick leave in addition to private insurance short term disability options to reduce financial constraints for patients (https://www.canada.ca/en/services/benefits/ei.html, https://www.dol.gov/whd/fmla/index.html, https://www.gov.uk/statutory-sick-pay).

There is limited literature on modern, real-world treatment timelines. We propose to determine the real world duration of curative treatment for the four most common cancers including recovery time between modalities. The goal is to understand the true length of curative intent cancer treatment to provide better guidance for the patient experience to inform their choices around social and family supports as well as workplace arrangements.

## Methods

### Database and inclusion/exclusion criteria

BC Cancer is the comprehensive cancer control program that serves a population of 5.07 million with 6 centres offering medical and radiation oncology services and a network of community oncology clinic sites for systemic therapy. It oversees the cancer registry that records all malignancies in the province and reports to the Canadian Cancer Registry. BC Cancer is the single payer for all systemic therapy and radiotherapy in the province. The 6 affiliated centres utilize the Cancer Agency Information system (CAIS), a common electronic medical record system.

A retrospective review was completed of patients from January 2010 to December 2016 referred to BC Cancer with newly diagnosed stage I-III breast, colorectal, NSCLC, or prostate cancer who received definitive, curative intent treatment with surgery and/or radiation. Patients ≤65 years old were included to reflect a working population. Patients were excluded from the study if they died less than 6 months from the time of diagnosis to avoid under-representing the duration of curative intent treatment or had received part of their treatment outside BC (wherein records were unavailable). The cohort was derived from the BC Cancer registry using diagnosis date, pathology and collaborative staging. The billing and treatment administration databases were used to abstract the chemotherapy type and dates. All radiotherapy is delivered at one of the 6 provincial centres and the dates were retrieved from the EMR. Missing data was abstracted by manual chart review.

### Timeline calculations and definitions

Breast cancer treatment included neoadjuvant/adjuvant chemotherapy and/or neoadjuvant/adjuvant radiation with definitive surgery. Hormone therapy and single agent trastuzumab delivery were excluded from timeline calculations. Colorectal cancer treatment included neoadjuvant/adjuvant chemotherapy and/or neoadjuvant/adjuvant radiation with definitive surgery. NSCLC cancer treatment included neoadjuvant/adjuvant chemotherapy and/or neoadjuvant/adjuvant/definitive radiation and/or definitive surgery. Prostate cancer treatment included definitive surgery and/or adjuvant/definitive radiotherapy. Radiotherapy was deemed adjuvant if it was delivered within 12 weeks of surgery. Hormone therapy duration was excluded.

Duration of a treatment modality was calculated for chemotherapy from the first dose of chemotherapy delivered to the last dose delivered. Radiotherapy treatment duration was calculated from the first fraction of radiotherapy to the last fraction of radiotherapy delivered. Combined modality chemoradiotherapy was calculated from the first radiation fraction or first chemotherapy dose to last radiation fraction or chemotherapy dose, whichever timepoint was later. These times were inclusive of weekends, holidays and other non-treatment days.

Treatment breaks or time between treatments were defined as the time between treatment modalities. Examples of treatment breaks include from surgery date to start of chemotherapy or from end of chemotherapy to start of radiotherapy. Treatment breaks were calculated using dates derived from respective treatment courses in the billing and treatment databases.

### Statistical analysis

Descriptive statistics were conducted using SPSS Version 26. Median treatment duration was defined as time from beginning of first treatment to end of last treatment including treatment breaks between different modalities. Each tumor specific cohort was further divided based on type of treatment, such as surgery alone versus multimodality treatment with the addition of neoadjuvant and/or adjuvant treatment.

Total treatment time was calculated for all patients who underwent multi-modality therapy. Overall the total treatment time began with the first cancer treatment to the last cancer treatment including intervals of recovery between modalities. The treatment time did not include recovery time after the completion of the last therapy.

The median total treatment time for each tumor group was weighted by the number of patients who received the specified course of treatment.

### Ethics statement

This retrospective chart review study involving human participants was in accordance with the ethical standards of the institutional and national research committee and with the 1964 Helsinki Declaration and its later amendments or comparable ethical standards. This study received approval from the University of British Columbia BC Cancer Agency Research Ethics Board (REB), H18–03294. There was no active enrollment or active follow-up of study subjects, and no data were collected directly from individuals. The researchers confirmed that they would take appropriate measures to protect the privacy of individuals, and to safeguard the identifiable information; comply with any known preferences previously expressed by individuals about any use of their information; acknowledged that it was impracticable to seek consent from individuals to whom the information relates; and obtained necessary permission for secondary use of information for research purposes to receive a waiver of consent from the REB for our retrospective study.

## Results

### Baseline characteristics

A total of 22,275 patients were referred to BC Cancer between January 2010 and December 2016 for curative intent treatment with 12,415 (55.7%) breast, 4988 (22.4%) colorectal, 2044 (9.2%) NSCLC, and 2828 (12.7%) prostate cancer patients. In the breast cancer cohort the median age was 55, 99.5% were female and the stage distribution was I/II/II 47.5/38.7/14.1% respectively. In the colorectal cancer cohort the median age was 58, 43.5% were female and the stage distribution was I/II/II 26.5/30.1/43.4% respectively. In the NSCLC cohort the median age was 60, 54% were female and the stage distribution was I/II/II 46.5/18.1/35.4% respectively. In the prostate cohort the median age was 61 and the stage distribution was I/II/II 7.7/62.9/29.4%. Baseline characteristics of each of the four cohorts are presented in Table 1.

**Table 1:** Baseline patient characteristics, *n* = 22,275

	Breast ***n*** = 12,415 (55.7%)	Colorectal ***n*** = 4988 (22.4%)	NSCLC ***n*** = 2044 (9.2%)	Prostate ***n*** = 2828 (12.7%)
Median age, years (IQR)	55 (48–60)	58 (52–62)	60 (55–63)	61 (57–63)
Sex				
Female (%)	12,357 (99.5)	2172 (43.5)	1103 (54.0)	N/A
Male (%)	58 (0. 5)	2816 (56.5)	941 (46.0)	2828 (100)
Stage				
I (%)	5856 (47.2)	1321 (26.5)	950 (46.5)	217 (7.7)
II (%)	4803 (38.7)	1499 (30.1)	370 (18.1)	1779 (62.9)
III (%)	1756 (14.1)	2168 (43.4)	724 (35.4)	832 (29.4)

Abbreviations: *IQR* interquartile range; *NSCLC* non-small cell lung cancer

### Breast cancer

The majority of breast cancer patients started therapy within 5 weeks of diagnosis (Table 2). The distribution of treatment strategies was 35.9% surgery alone, 19.8% surgery and radiotherapy, 25.9% surgery and chemotherapy and 18.4% surgery with radiation and chemotherapy. Overall 64.1% of patients received multi-modality therapy. Chemotherapy was delivered in 5490 (69%) of the 7952 of multimodality patients and radiotherapy in 4743 (60%). The median duration of chemotherapy ranged from 14 to 15 weeks and radiotherapy 3.6–5.4 weeks. Depending on the sequencing of therapy the median recovery period between modalities ranged from 4.6–12.1 weeks. For protocols that required three modalities of treatment the median recovery time between treatments was up to 13.9 weeks. Median total treatment time for the multimodality breast cohort was 24.6 weeks (IQR 17.0–32.3). Duration of therapy was examined by stage, year of diagnosis and median age for breast cancer (Table 3). This demonstrated that more advanced stage, age younger than the median were associated with longer duration of treatment. There was no consistent trend by year of therapy.

**Table 2:** Median duration of multimodality cancer treatments in weeks

	n (%)	Diagnosis to first treatment (IQR)	Chemotherapy (IQR)	Radiation (IQR)	Time between treatments (1) (IQR)^**b**^	Time between treatments (2) (IQR)^**b**^	Overall cancer treatment (IQR)
**BREAST,** ***n*** **= 12,415**							
**Surgery Only**	4463 (35.9)	5.7 (3.9–8.1)					
**Surgery + Radiotherapy**							
Neoadjuvant XRT	26 (0.2)	29.1 (25.9–32.5)		5.4 (4.5–5.7)	6.9 (5.1–9.0)		11.9 (10.4–14.3)
Adjuvant XRT	2436 (19.6)	5.0 (3.7–7.1)		3.6 (3.1–4.3)	12.1 (9.6–18.1)		16.0 (13.3–22.4)
**Surgery + Chemotherapy**							
Neoadjuvant Chemo	495 (4.0)	5.0 (3.9–7.9)	15.0 (12.1–20.9)		6.1 (4.4–12.9)		23.9 (19.4–28.0)
Adjuvant Chemo	2714 (21.9)	5. 0 (3.6–6.6)	14.3 (9.0–20.6)		8.0 (6.3–10.3)		22.4 (18.1–28.1)
**Surgery + Chemotherapy + Radiotherapy**							
Surgery > > Adj Chemo > > Adj XRT	1895 (15.3)	4.9 (3.4–6.3)	14.0 (9.0–16.1)	4.0 (3.1–5.4)	8.3 (6.6–10.9)	5.0 (4.0–8.3)	33.9 (29.3–38.6)
Chemo > > Surgery > > Adj XRT	310 (2.5)	5.0 (3.4–8.0)	14.5 (9.0–19.8)	5.3 (3.4–5.7)	6.1 (4.4–10.3)	7.8 (6.1–10.2)	35.7 (31.7–40.9)
Chemo > > XRT > > Surgery	76 (0.6)	5.6 (3.6–13.8)	15.0 (9.0–21.7)	5.4 (4.0–5.9)	4.6 (3.6–7.7)	6.9 (5.3–9.0)	35.3 (27.9–40.0)
**Total Treatment Time**^a^	**7952 (64.1)**						**24.6 (17.0–32.3)**
**COLORECTAL,** ***n*** **= 4988**							
**Surgery Only**	2895 (58.0)	3.0 (0.0–6.3)					
**Surgery + Radiotherapy**							
Neoadjuvant XRT	116 (2.3)	7.9 (6.0–9.9)		0.6 (0.6–0.9)	1.2 (0.9–2.0)		2.0 (1.4–2.6)
Adjuvant XRT	23 (0.5)	3.9 (0.0–7.4)		5.0 (3.9–5.4)	9.0 (6.9–11.7)		14.4 (10.9–17.1)
**Surgery + Chemotherapy**							
Neoadjuvant Chemo	68 (1.4)	6.1 (4.3–8.0)	5.1 (4.0–5.9)		8.9 (7.3–10.7)		13.9 (12.3–15.1)
Adjuvant Chemo	1501 (30.1)	4.0 (0.4–8.1)	20.9 (9.1–22.6)		8.3 (6.7–11.4)		28.7 (22.1–32.1)
**Surgery + Chemotherapy + Radiotherapy**							
Surgery > > ChemoXRT	84 (1.7)	3.5 (0.1–7.3)	14.0 (5.9–18.5)		8.9 (6.9–13.4)		29.6 (14.9–34.3)
ChemoXRT > > Surgery	170 (3.4)	7.5 (6.1–9.5)	6.0 (5.0–14.0)		7.4 (6.1–8.7)		14.0 (12.0–15.9)
XRT > > Surgery > > Adj Chemo	67 (1.3)	8.00 (6.3–10.0)	21.0 (5.4–23.0)	0.9 (0.6–0.9)	1.1 (0.7–1.7)	9.7 (7.1–11.9)	31.0 (24.3–35.3)
ChemoXRT > > Surgery > > Chemo	64 (1.3)	7.3 (5.9–9.9)	34.6 (29.9–37.4)				34.6 (29.9–37.4)
**Total Treatment Time**^a^	**2093 (42.0)**						**26.7 (14.3–31.4)**
**LUNG,** ***n*** **= 2044**							
**Surgery Only**	1067 (52.2)	0.0 (0.0–6.7)					
**Radiotherapy Only**	190 (9.3)	11.1 (6.8–17.4)		3.2 (1.4–5.9)			3.2 (1.4–5.9)
**Sequential Chemo > > Radiotherapy**	92 (4.5)	8.9 (4.5–13.7)	9.3 (6.3–10.3)	5.4 (3.0–6.1)	6.3 (1.0–9.3)		19.1 (10.9–25.0)
**Combined Chemoradiotherapy**	421 (20.6)	6.7 (4.2–9.1)	6.9 (6.0–10.3)				6.9 (6.0–10.3)
**Surgery + Radiotherapy**							
Neoadjuvant XRT	4 (0.2)	7.4 (5.8–11.7)		6.0 (5.3–7.0)	10.0 (4.8–21.3)		16.6 (10.0–27.6)
Adjuvant XRT	6 (0.3)	8.1 (0.0–12.8)		1.9 (1.3–4.0)	13.3 (11.6–16.4)		15.8 (12.8–21.3)
**Surgery + Chemotherapy**							
Adjuvant Chemo	214 (10.5)	5.9 (0.0–9.9)	10.0 (6.4–11.0)		8.6 (7.3–10.0)		18.4 (15.1–20.8)
**Surgery + Chemotherapy + Radiotherapy**							
ChemoXRT > > Surgery	29 (1.4)	7.6 (5.9–10.1)	6.1 (5.2–9.3)		7.4 (5.7–13.7)		18.0 (11.1–29.3)
Surgery > > ChemoXRT	21 (1.0)	4.3 (0.0–8.4)	9.9 (5.8–11.6)		8.0 (6.4–11.4)		26.6 (18.7–35.6)
**Total Treatment Time**^a^	**977 (47.8)**						**9.1 (6.0–16.3)**
**PROSTATE,** ***n*** **= 2828**							
**Surgery Only**	1134 (40.1)	13.0 (9.4–18.8)					
**Definitive Radiotherapy Only**	654 (23.1)	33.1 (24.0–43.1)		5.9 (4.9–7.6)			5.9 (4.9–7.6)
**Brachytherapy**	1014 (35.9)	25.3 (18.9–34.9)					
**Surgery + Radiotherapy**							
Adjuvant XRT (within 3 months)	26 (0.9)	9.2 (6.5–15.8)		6.7 (6.5–7.0)	10.0 (9.3–11.5)		17.1 (16.1–18.1)
**Total Treatment Time**^a^	**680 (24.0)**						**6.0 (4.9–7.6)**

Abbreviations: *Adj* adjuvant; *XRT* radiotherapy; *IQR* interquartile range

^a^Total treatment time – median duration calculated from all *multimodality* treatment cases (i.e. excludes cases of surgery/brachytherapy alone)

^b^Time between treatments – median duration calculated from the date of the last treatment of the first modality to the date of the first treatment of the next modality

**Table 3:** Median duration of multi-modality treatment by stage, year of diagnosis and median age

	Median duration of multi-modality therapy in weeks (IQR)	*p*-value
**Breast**		
Stage		
I	17.6 (14.0–25.0)	< 0.001
II	27.6 (20.3–34.0)	
III	30.4 (22.7–36.1)	
Year of diagnosis		
2010–11	25.3 (15.7–33.3)	< 0.001
2012–13	24.3 (16.6–32.3)	
2014–15	23.3 (18.9–29.3)	
2016	28.0 (18.6–36.8)	
Age		
< = 55 (the median)	26.0 (18.4–33.1)	< 0.001
> 55	22.1 (15.7–31.3)	
**Colorectal**		
Stage		
I	14.3 (4.5–25.3)	< 0.001
II	22.1 (12.4–30.4)	
III	27.9 (16.0–31.9)	
Year of diagnosis		
2010–11	16.3 (11.7–30.1)	< 0.001
2012–13	27.6 (14.7–31.6)	
2014–15	28.9 (21.9–32.7)	
2016	20.3 (13.5–28.1)	
Age		
< = 58 (the median)	26.3 (14.8–31.0)	0.18
> 58	27.6 (14.0–32.0)	
**NSCLC**		
Stage		
I	4.1 (1.4–14.5)	< 0.001
II	16.3 (9.0–19.7)	
III	7.3 (6.0–12.4)	
Year of diagnosis		
2010–11	7.0 (5.8–15.8)	0.002
2012–13	10.1 (6.0–17.0)	
2014–15	9.3 (6.0–17.4)	
2016	6.7 (6.0–14.5)	
Age		
< = 60 (the median)	10.1 (6.0–17.4)	0.21
> 60	7.0 (5.8–14.2)	
**Prostate**		
Stage		< 0.001
I	7.9 (6.9–8.1)	
II	7.3 (5.4–7.7)	
III	7.4 (7.1–7.9)	
Year of diagnosis		
2010–11	7.3 (5.7–7.7)	0.29
2012–13	7.3 (5.4–7.7)	
2014–15	7.4 (6.0–7.9)	
2016	7.4 (7.0–7.9)	
Age		
< = 61 (the median)	7.3 (5.4–7.9)	0.57
> 61	7.4 (5.0–7.7)	

Abbreviations: *IQR* interquartile range

### Colorectal cancer

Of the 4988 patients, 58.0% received definitive surgery alone, 2.8% surgery and radiotherapy, 31.5% surgery and chemotherapy and 7.7% surgery with radiotherapy and chemotherapy (Table 2). Median diagnosis to initiation of therapy time was 3–8 weeks. Chemotherapy was incorporated in the treatment regimen of 1954 (93%) patients and radiotherapy in 524 (25%) of the 2093 multimodality treated patients. The median duration of chemotherapy ranged from 5.1–21 weeks, concurrent chemoradiotherapy 6–14 weeks and radiotherapy 0.6–5 weeks. The median time between treatments ranged from 1.1–9.7 weeks with the shorter time intervals noted between neoadjuvant radiotherapy and surgery. The median total treatment time for multimodality treatment was 26.7 weeks (IQR 14.3–31.4). Therapy duration was examined by stage, year of diagnosis and median age for colorectal cancer (Table 3). More advanced stage was associated with longer treatment times however, there was no difference based on median age and no consistent trend by year of treatment.

### Non-small cell lung cancer

There were 52.2% of patients who received definitive surgery alone, 9.3% received definitive radiotherapy alone, 25.1% who received chemotherapy and definitive radiotherapy, 0.5% surgery and radiotherapy, 10.5% surgery and chemotherapy and 2.4% surgery, chemotherapy and radiation treatment (Table 2). The median time from diagnosis to first treatment was 0–11.1 weeks as some patients received a definitive diagnosis at the time of surgical resection. Chemotherapy median duration ranged from 6.1–10 weeks with shorter treatment courses when delivered concurrently with radiotherapy. Definitive radiotherapy median treatment time ranged from 3.2 weeks as a single modality to 6.9 weeks concurrently with chemotherapy and neoadjuvant/adjuvant median durations of 1.9–6 weeks. Median recovery time between treatment modalities was 6.3–13.3 weeks. The median total multimodality treatment time was 9.1 weeks (IQR 6.0–16.3). The NSCLC treatment time was evaluated by stage, year of diagnosis and median age (Table 3). Stage II had the longest duration of treatment (adjuvant chemotherapy) followed by stage III (radiotherapy +/− chemotherapy). There was no consistent trend by year and no difference by median age.

### Prostate cancer

The median age of prostate cancer diagnosis was 61 years (Table 1). The majority of patients received definitive surgery only 1134 (40.1%) or brachytherapy only 1014 (35.9%) (Table 2). Definitive radiotherapy was delivered to 23.1% and adjuvant radiotherapy 0.9% with a median duration of 5.9 and 6.7 weeks respectively. The median time between surgery and adjuvant radiotherapy was 10 weeks. For those patients treated with radiotherapy or surgery with adjuvant radiotherapy, median total treatment time was 6.0 weeks (IQR 4.9–7.6 weeks) (Table 2).

Prostate cancer duration of therapy was assessed by stage, year of diagnosis and median age (Table 3). Stage resulted a statistically significant different in median duration of treatment that was not clinically significant. Median age and year of treatment did not impact duration of treatment.

## Discussion

With the evolution of cancer care, overall cancer mortality rates have decreased [8]. In our study of the four most common cancers, the real world median duration of treatment varied significantly depending on tumor site and treatment modality. Patients had between treatment periods of up to 10 weeks, significantly contributing to the total therapy time. Notably breast and colorectal cancer, the largest patient populations, had the longest multimodality treatment durations of 24.6 and 26.7 weeks respectively. Cancer treatment and recovery can cause significant prolonged disruptions in patient’s life and plans that needs to be acknowledged to enable successful transition to life in the post-treatment phase.

The standard duration of adjuvant chemotherapy has been well outlined in guidelines. For breast cancer, typical regimens involve 6–8 cycles of chemotherapy on a 3 week schedule or a dose dense 2 week schedule [9, 10]. Our study noted median durations of 14–15 weeks for chemotherapy consistent with these recommendations. With colorectal cancer the duration of adjuvant oxaliplatin based chemotherapy for 3 or 6 months is dependent on the risk profile of stage III disease [4]. For high risk stage II disease, consideration of adjuvant therapy is based on disease and patient characteristics and risk profile [11]. The standard chemotherapy duration in our study had an interquartile range from 9.1–22.6 weeks suggesting that risk based assessment of adjuvant treatment duration was employed in our province. Similarly, in resected NSCLC the guidelines recommended 4 cycles every 3 weeks of adjuvant platinum based chemotherapy, corresponded to the observed 10 week median in our study [5]. The correlation between the duration of treatment in the guidelines and our study suggests that treatment in BC is consistent with current recommendations and accurately reflects the cancer treatment timeline experience for many patients.

Neoadjuvant, adjuvant and definitive curative intent radiotherapy standards are well-established for best practice. With breast cancer, the recommended adjuvant whole breast irradiation program with breast conserving surgery is 40–42.5 Gray in 15–16 fractions [12]. Our study noted a median duration of treatment of 3.6 weeks, consistent with these recommendations. In our colorectal cohort, a select group of patients received radiotherapy, predominantly those with rectal cancer [13]. Short course neoadjuvant radiotherapy (9%) and neoadjuvant chemoradiotherapy (11%) were common strategies for patients who received multimodality therapy. Definitive radiotherapy in NSCLC may include stereotactic body radiotherapy for early stage staged disease and standard fractionation with or without chemotherapy for locally advanced disease to 60 Gray [14, 15]. These potential strategies were reflected in the median duration of therapy noted in our study. Prostate cancer radiotherapy can be delivered with standard fractionation or hypofractionated external beam radiotherapy typically for 4–8 weeks [16]. The median duration of prostate cancer treatment in BC was 6 weeks. Across all tumor sites, similar to chemotherapy, the duration of radiation therapy for the difference disease sites in our study were consistent with the expected timelines from clinical trials data suggesting our results are generalizable to other populations.

While the expected duration of chemotherapy and radiotherapy can be estimated, there is less clarity around time between treatment modalities. This non-treatment time may account for healing post-surgery, recovering from the side effects of chemotherapy and/or radiotherapy and reflects the toxicity associated with cancer treatments. Other factors including delays in access to treatments due to resource constraints may also contribute to this time. Our dataset did not have sufficient granularity to examine the respective reasons for the timing gaps. During these times, patients are not on active treatment and yet are not well enough to resume normal life activities as they regain their functional status for the next type of treatment or are not eligible to return to the workplace due to the next expected treatment. Patients may spend a significant amount of time obtaining clinical services. One study demonstrated that over half of clinical visits were spent on commuting and waiting for care [17]. Time between treatment modalities may be spent on the preparation and planning of the next phase of therapy. Our analysis highlights that there is a wide variety in time between treatment modalities in the real world, up to 13–14 weeks in patients who require all three modalities of therapy. As many studies focus of the specified treatment modality, this aspect of recovery is often not captured well in clinical trials. In addition, after the completion of all modalities of treatment patients also require a recovery period that was not accounted for in our study because there was no consistent time point available to calculate this aspect.

Recognizing that from start to finish, curative intent cancer treatments can extend to over 6 months, without considering the final post treatment recover phase, the implications for life disruption can be significant. In addition to the need for social supports, this duration of treatment can cause significant financial distress due to loss of income and the out of pocket costs of cancer care. Many countries have laws and social structures that try to provide workplace protection and financial support for cancer patients. In Canada, taxpayers are offered 15 weeks employment insurance coverage for illness or as a caregiver for a family member through government policies and the majority of Canadians have a component of private insurance typically through workplace coverage (https://www.canada.ca/en/services/benefits/ei.html [18]) In the United States (US) the Family and Medical Leave Act entitles eligible employees of covered employers to take unpaid, job-protected leave with continuation of group health insurance coverage for up to 12 weeks (https://www.dol.gov/whd/fmla/index.html). US census data from 2018 indicate that 67.3% of individuals also have private insurance through an employer or union that may provide disability coverage [19]. The United Kingdom’s Statutory Sick Pay provides financial support to employees for up to 28 weeks with a small proportion of private insurance coverage, 10.5% (https://www.gov.uk/statutory-sick-pay [23]). Patient access to employment benefits such as paid sick leave, extended sick leave, or even unpaid leave is associated with a reduction in personal financial burden for the individual and job retention during illness [20]. In Canada, the need for longer financial assistance has been recognized and advocacy to extend coverage through revision of the Employment Insurance Act is pending [21].

Predicting who may require longer treatment and recovery durations may be helpful for patient life planning. Across breast, colorectal and NSCLC cancer, higher stage was associated with long treatment times as additional modalities and treatment regimens may have been added for risk reduction. Age was a relevant factor for breast cancer treatment however, it did not impact the other cancer types. Over the 7-year time period from 2010 to 2016 there was not any clear trends in duration of therapy. While advances in systemic therapy have occurred during the time interval this may have been due to sequencing or addition of new therapeutic agents rather than lengthening the treatment course.

The limitations of our study include its retrospective design, lack of employment status, inability to collect out of pocket treatment recommendation compliance and utilization of government and/or workplace supports within our cohort. It is also noted that our treatment duration only incorporated active treatment and breaks between modalities, but did not include the final post-treatment recovery time. Similarly, our findings do not characterize the variations in treatment regimen, for example, there would likely be differences in feasibility of employment based on scheduling (radiotherapy daily versus chemotherapy every 3–4 weeks), delivery method (intravenous versus oral) and side effects. Our strengths include the population-based dataset that reflects a real-world cancer survivor cohort and the large number of patients included in this study.

## Conclusions

In conclusion, approximately half of patients who receive curative intent treatment require multimodality treatment with total duration as long as 26.7 weeks. The time between treatment modalities contributed significantly to the overall timeline ranging up to 13–14 weeks for patients requiring surgery, radiotherapy and chemotherapy. The inter-treatment duration contributes significantly to the total length of adjuvant treatment. The results of our study demonstrate that the durations of modern curative intent treatments and recovery can be prolonged for cancer patients. Patient awareness of the potential timelines may help with life and workplace organization and planning. Government supported programs should provide flexibility for extended employment protection and financial assistance to support cancer patients on their journey.

## Data Availability

The datasets generated during and analysed during the current study are available from the corresponding author on reasonable request.

## Abbreviations

Adj: Adjuvant
BC: British Columbia
CAIS: Cancer Agency Information System
IQR: Interquartile range
NSCLC: Non small cell lung cancer
REB: Research ethics board
US: United States
XRT: Radiotherapy
